# Human papillomavirus genotypes and the risk factors associated with multicentric intraepithelial lesions of the lower genital tract: a retrospective study

**DOI:** 10.1186/s12879-021-06234-0

**Published:** 2021-06-11

**Authors:** Jing Zhang, Guangcong Liu, Xiaoli Cui, Huihui Yu, Danbo Wang

**Affiliations:** 1 Department of Gynecology, Cancer Hospital of China Medical University, Liaoning Cancer Hospital and Institute, No.44 Xiaoheyan Road, Shenyang, 110042 Liaoning province China; 2 Department of Epidemiology, Cancer Hospital of China Medical University, Liaoning Cancer Hospital and Institute, Shenyang, 110042 Liaoning province China; 3 Department of Cancer Prevention and Treatment, Cancer Hospital of China Medical University, Liaoning Cancer Hospital and Institute, Shenyang, 110042 Liaoning province China

**Keywords:** Multicentric intraepithelial lesions, Human papillomavirus, Genotypes, Prevalence, Risk factors

## Abstract

**Background:**

Multicentric intraepithelial lesions of the lower genital tract (multicentric lesions) were defined as intraepithelial lesions of two or three sites within cervix, vagina, and vulva occurring synchronously or sequentially. The characteristics of multicentric lesions has been poorly understood. This study aimed to evaluate the risk factors for multicentric lesions, including specific HPV genotypes.

**Methods:**

A retrospective case-control study was performed involving patients histologically diagnosed with multicentric lesions between January 2018 and October 2019. Controls were patients histologically diagnosed with single cervical intraepithelial neoplasia (CIN) and admitted during the same period. Univariable and multivariable analyses were used to assess the risk factors for multicentric lesions.

**Results:**

Of 307 patients with multicentric lesions, the median age was 50 years (interquartile range: 43–55.5), and they were older than patients with single CIN (median age: 43 years, interquartile range: 36–50). In the multicentric lesion group, the proportions of cytologic abnormalities, HPV positivity, and multiple HPV infections were 68.9, 97.0, and 36.5%, respectively. In the multivariable analysis, menopause, a history of malignant tumors beyond the lower genital tract and multiple HPV infections were associated with the incidence of multicentric lesions (Odd ratio (OR) = 3.14, 95% confidence interval (CI) 2.24–4.41; OR = 9.58, 95% CI 1.02–89.84; OR = 1.47, 95% CI 1.03–2.10). The common HPV genotypes were HPV16, HPV53, HPV58, HPV52, HPV51, HPV56 and HPV18 in patients with multicentric lesions. The proportion of HPV16 infection was higher in high-grade lesions group than that in low-grade lesions group (OR = 2.54, 95% CI 1.34–4.83). The OR for multicentric lesions, adjusted for menopause, smoking, gravidity, parity, a history of malignant tumor beyond the lower genital tract and multiple HPV infection, was 1.97 (95% CI 1.04–3.75) in patients with HPV51 infection.

**Conclusions:**

Multicentric lesions were associated with menopause, a history of malignant tumors and multiple HPV infections. HPV16 was the most common genotype, especially in high grade multicentric lesions and HPV51 infection was found to be a risk factor for detecting multicentric lesions.

## Background

Persistent human papillomavirus (HPV) infections play a critical role in the development of lower genital tract precancerous and cancerous diseases. Cervical intraepithelial neoplasia (CIN) is common with an incidence that is ten times higher than that of vaginal intraepithelial neoplasia (VAIN) or vulvar intraepithelial neoplasia (VIN). However, the incidence of VAIN or VIN has increased recently [1]. In 1960, Marcus et al. [2] first described the theory of multicentric origin in lower genital tract. Multicentric intraepithelial lesions of the lower genital tract (referred to as multicentric lesions) were defined as those occurring in two or three sites within the cervix, vagina, and vulva, which could occur synchronously or sequentially [3]. Multicentric lesions can occur sequentially but be detected at the same clinical visit [4]. Multicentric lesions were significantly more likely to be detected synchronously than sequentially [5].

A comprehensive diagnosis of multicentric lesions can be challenging, as vaginal or vulvar lesions are easily missed on clinical exam; these missed diagnoses lead to a poor prognosis. Previous studies have found that multicentric lesions are a risk factor for residual lesion and disease recurrence [3, 6, 7]. At present, there are few articles which describing the characteristics and significance of multicentric intraepithelial lesions. and the sample sizes of the previous studies have been small [3–5]. Moreover, HPV testing is useful for the detection of multicentric lesions, however, available data about the HPV genotypes prevalence in multicentric lesions are limited.

Therefore, in this study, we aimed to describe the clinical characteristics of a large number of patients with multicentric intraepithelial lesions of the lower genital tract which were detected synchronously. We also examined the prevalence of different HPV genotypes. Correlations between several risk factors and the incidence of multicentric lesions were also assessed.

## Methods

### Study population

A retrospective case-control study was conducted with patients undergoing colposcopy-guided biopsy at the Cervical Disease Clinic of Liaoning Cancer Hospital and Institute, which serves as a referral center in northeastern China. Patients with a histologically confirmed diagnosis of multicentric lesions detected synchronously (CIN with VAIN and/or VIN, VAIN with VIN), between January 1st, 2018, and October 31st, 2019, were included in the study and were placed into the multicentric lesions group. Moreover, we enrolled patients with histologically confirmed diagnoses of single CIN, who were admitted during the same period to be in the single CIN control group. Exclusion criteria were the presence of concurrent invasive cancer in the lower genital tract, a history of invasive cancer in the lower genital tract, multicentric lesions detected sequentially or pregnancy. In the multicentric group, two subgroups were divided according to the grade of CIN, including high-grade lesions group (CIN2+ with VAIN and/or VIN) and low-grade lesions group (CIN1 with VAIN and/or VIN).

### Clinical data

Clinical data were retrieved retrospectively from electronic medical records and picture archiving and communication systems (PACS) in the hospital by trained gynecological staff using standardized data collection and quality control procedures, including demographic details, menopause status, symptoms, a history of other diseases, cytology results (identified with ThinPrep® liquid-based cytology system, Hologic Inc., MA, USA), HPV infection and genotypes, high-risk HPV (HR-HPV) viral loads and histopathologic results.

HR-HPV DNA viral loads were detected using the Digene Hybrid Capture 2 (HC2) test (Digene Co., MD, USA). HPV DNA was detected and genotyped with the HPV GenoArray test kit (Hybribio Ltd., Hong Kong) using both DNA amplification and a flow-through hybridization technique [8]. A total of 21 genotypes were screened, including13 high-risk genotypes (HPV16, HPV18, HPV31, HPV33, HPV35, HPV39, HPV45, HPV51, HPV52, HPV56, HPV58, HPV59, and HPV68), two probable high-risk genotypes (HPV53 and HPV66) and six low-risk genotypes (HPV6, HPV11, HPV42, HPV43, HPV44, and CP8304).

Colposcopy was performed in the Cervical Disease Clinic of our hospital. Women were referred to colposcopy if cytologic result was low-grade squamous intraepithelial lesion (LSIL) or worse, or cytologic result was atypical squamous cell of unknown significance (ASCUS) companied with HPV infection, or HPV16/18 were positive with normal cytology, or other HR-HPV genotype infection persists 6 months with normal cytology. Comprehensive assessments of the cervix, vagina, and vulva were carried out. Colposcopy-guided biopsies were performed if suspicious lesions were found. Biopsy tissue sections were independently assessed by two expert pathologists.

### Statistical analyses

Normality of continuous variables was determined using the Kolmogorov-Smirnov test. When data were continuously and normally distributed, quantitative variables were presented as the mean ± standard deviation (SD), and the Student’s *t*-test was used to compare differences. When data were abnormally distributed, quantitative variables were presented as the Median and Interquartile range (IQR), and the Mann-Whitney U test was used to compare differences. Univariable analyses were performed to quantify the association between independent variables and binary outcomes using logistic regression analyses for categorical variables. The multiple logistic regression model was used to quantify independent factors associated with multicentric lesions. The prevalence of HPV was expressed as the proportion of HPV-positive cases compared with the total number of HPV cases. Logistic regression analyses, which were adjusted for menopause, a history of malignant tumors, and multiple HPV infections, were used to estimate correlations between specific HR-HPV genotypes and the number of multicentric lesions detected, and the Odds ratios (ORs) with 95% confidence intervals (CIs) were calculated. Data were analyzed using the SPSS version 22.0 software (SPSS Inc., Chicago, IL, USA).

This study was approved by the ethical committee of Liaoning Cancer Hospital and Institute (reference number 2020–0610); this approval allowed access to the data used for this research.

## Results

### Clinical characteristics

**Fig. 1:**
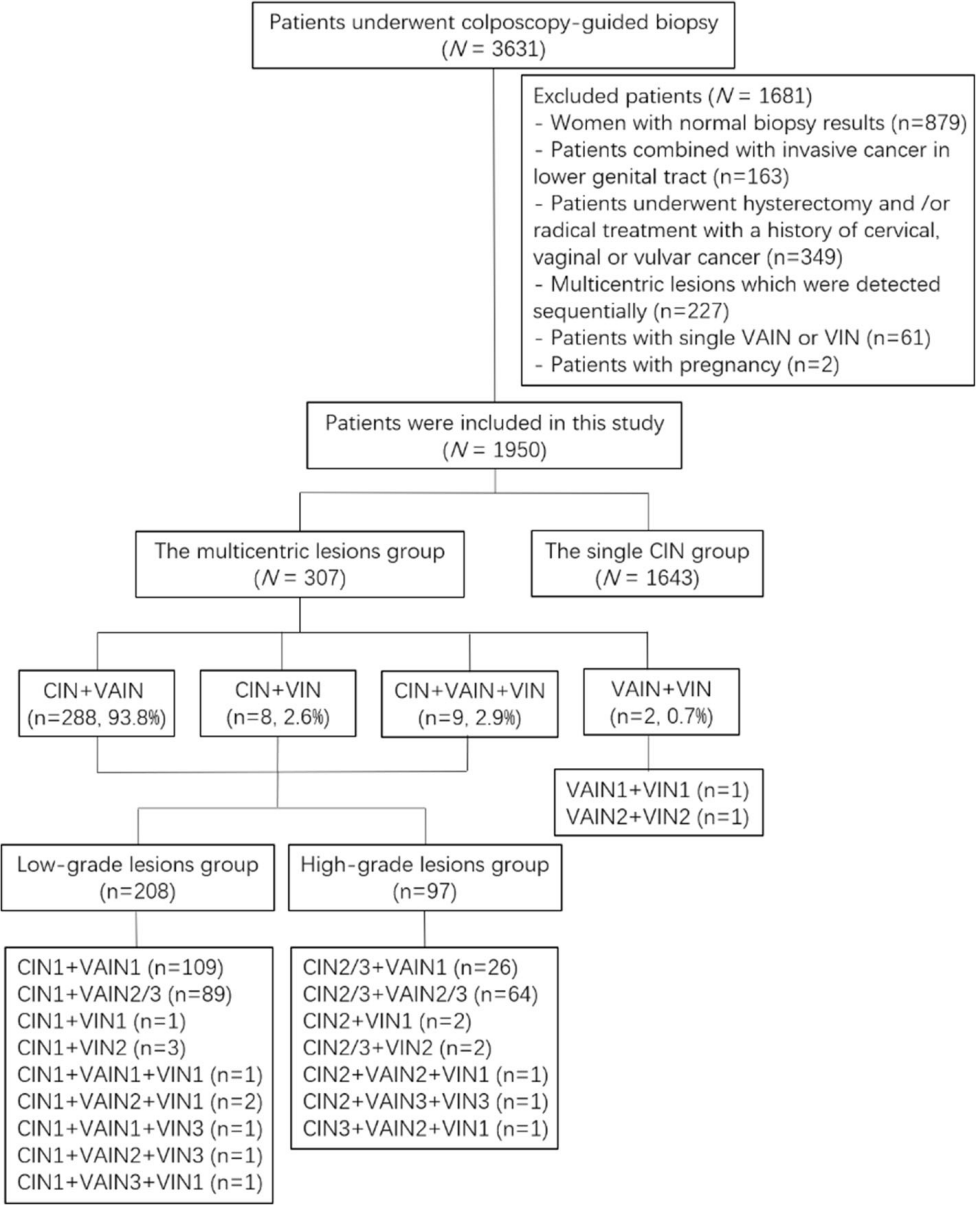
Flow diagram of the study population. (CIN, cervical intraepithelial neoplasia; VAIN, vaginal intraepithelial neoplasia; VIN, vulvar intraepithelial neoplasia)

A total of 1950 patients were enrolled, including 307 patients in the multicentric lesions group and 1643 patients in the single CIN group (Fig. 1). The clinical characteristics of the two groups are presented in Table 1. The median age of the multicentric lesions group was higher than that of the single CIN group (50 years, interquartile range 43–55.5; 43 years, interquartile range 36–50, respectively). In the multicentric lesions group, 279 patients (279/307, 90.9%) were asymptomatic. In addition, nine patients had postcoital bleeding, 8 patients had abnormal vaginal bleeding, 5 patients had vulvar itching, 5 patients had increased vaginal discharge and 1 patient had bleeding after menopause. In the multicentric lesions group, 13 patients had a history of other diseases (6 cases with breast cancer, one case with lung cancer, one case with lymphoma, one case with thyroid cancer, one case with endometrial cancer, one case with thrombocytopenia, one case with depression and one case with hypothyroidism). Ten (3.3%) patients had a history of malignant tumor beyond lower genital tract.

**Table 1:** Clinical characteristics of patients in both groups, univariable analysis and multivariable analysis: factors associated with multicentric lesions

Characteristics		The multicentric lesions group, *N* = 307	The single CIN group, *N* = 1643	Univariable analyses OR (95% CI)	Multivariable analyses OR (95% CI)
Age, median (Q1-Q3)		50 (43–55.5)	43 (36–50)		
Menopause, n (%)	Yes	158 (51.5)	460 (28.0)	2.73 (2.13–3.50)	3.14 (2.24–4.41)
	No	149 (48.5)	1183 (72.0)		
Smoking, n (%)	Yes	8 (2.6)	29 (1.8)	1.49 (0.67–3.29)	1.74 (0.59–5.14)
	No	299 (97.4)	1614 (98.2)		
Gravidity, n (%)	≤2	126 (41.0)	621 (37.8)	1.15 (0.89–1.47)	1.32 (0.92–1.90)
	≥3	181 (59.0)	1022 (62.2)		
Parity, n (%)	≤2	285 (92.8)	1531 (93.2)	0.95 (0.59–1.52)	0.92 (0.47–1.80)
	≥3	22 (7.2)	112 (6.8)		
A history of malignant tumor beyond the lower genital tract, n (%)	Yes	10 (3.3)	12 (0.7)	4.58 (1.96–10.69)	9.58(1.02–89.84)
	No	297 (96.7)	1631 (99.3)		
Cytology, n (%)	Abnormal	184 (68.9)	833 (62.5)	0.75 (0.57–1.00)	
	Normal	83 (31.1)	499 (37.5)		
HPV test, n (%)	Positive	294 (97.0)	1391 (94.1)	2.07 (1.03–4.15)	
	Negative	9 (3.0)	88 (5.9)		
Multiple HPV infection, n (%)	Yes	65 (36.5)	223 (27.2)	1.54 (1.09–2.17)	1.47(1.03–2.10)
	No	113 (63.5)	597 (72.8)		
HR-HPV viral load, median (IQR)		272.165 (1118.17)	10.13 (16.70)		
HR-HPV viral load, n (%)	>1000	27 (26.5)	64 (17.0)	1.76 (1.05–2.95)	
	≤1000	75 (73.5)	313 (83.0)		

*CIN* cervical intraepithelial neoplasia, *SD* standard deviation, *HPV* human papillomavirus, *HR-HPV* high-risk human papillomavirus, *OR* odds ratio, *CI* confidence interval, *IQR* Interquartile range

Cytologic results were available for 267 patients in the multicentric lesions group and 1332 patients in the single CIN group. Among them, 184 (68.9%) patients in the multicentric lesions group and 833 (62.5%) patients in the single CIN group had abnormal cytologic results, such as atypical squamous cell with unknown sense or worse (Table 1).

HPV status was available in 303 patients in the multicentric lesions group. Patients in the multicentric lesions group as compared to controls were more likely to be HPV positive (OR = 2.07, 95% CI 1.03–4.15). HR-HPV viral loads were available for 102 patients in the multicentric lesions group. The median relative light units over a positive control-derived cutoff value (RLU/CO) was 272.165 with an interquartile range (IQR) of 1118.17, which was higher than that in the single CIN group (10.13 RLU/CO, IQR:16.70) (Table 1).

### The prevalence of the HPV genotypes

In the multicentric lesions group, HPV genotype results were available for 177 patients. Multiple HPV infections were identified in 65/178, 36.5%. One patient in the multicentric lesions group and five patients in the single CIN group was recorded to have multiple HPV infection, but their HPV genotyping results were unavailable. The proportion of patients with multiple HPV infections in the multicentric lesions group was higher than in the single CIN group (OR = 1.54, 95%CI 1.09–2.17) (Table 1). The prevalence of HPV genotypes in the multicentric lesions and single CIN groups are shown in Table 2. HPV16 was the most common genotype in both groups. In the multicentric lesions group, HPV53 (24, 13.6%) ranked second, followed by HPV58 (22, 12.4%), HPV52 (19, 10.7%), HPV51 (17, 9.6%), HPV56 (17, 9.6%), and HPV18 (16, 9.0%). However, HPV52 ranked second in the single CIN group, whereas HPV18 ranked fourth, and HPV53 dropped to the sixth most common in this group. There were low-risk genotype infections in both groups. The common genotypes were HPV43, HPV81, HPV42, and HPV6. However, the low-risk genotypes were always present with high-risk genotypes as co-infections. Only two patients had HPV43 single infections in the multicentric lesions group.

**Table 2:** The prevalence of HPV genotypes in patients of both groups

HPV genotypes	The multicentric lesions group (*n* = 177)	The single CIN group (*n* = 815)
High-risk genotypes, n (%)		
16	69(39.0)	338(41.5)
18	16(9.0)	68(8.3)
31	11(6.2)	51(6.3)
33	15(8.5)	60(7.4)
35	3(1.7)	24(2.9)
39	3(1.7)	34(4.2)
45	6(3.4)	6(0.7)
51	17(9.6)	35(4.3)
52	19(10.7)	123(15.1)
53	24(13.6)	51(6.3)
56	17(9.6)	45(5.5)
58	22(12.4)	115(14.1)
59	6(3.4)	23(2.8)
66	4(2.3)	29(3.6)
68	9(5.1)	41(5.0)
Low-risk genotypes, n (%)		
6	5(2.8)	10(1.2)
11	3(1.7)	1(0.1)
42	5(2.8)	20(2.5)
43	7(4.0)	10(1.2)
44	1(0.6)	2(0.2)
81	6(3.4)	19(2.3)

Note: Women with multiple HPV types detected are counted to each type, and therefore counted more than once

*HPV* human papillomavirus, *CIN* cervical intraepithelial neoplasia

### Comparison between two subgroups of the multicentric lesion group

The median age of patient in both subgroups was similar (Low-grade lesions group median age: 50 years, Q1-Q3: 43–56; High-grade lesions group median age: 50 years, Q1-Q3: 41.5–55). If the cervical intraepithelial lesions were high grade, combined vaginal or vulvar intraepithelial lesions was more likely to be high grade (OR = 2.82, 95%CI 1.68–4.73). Compared to low-grade lesions group, HPV16 infection was more popular in high-grade lesions group (OR = 2.54, 95%CI 1.34–4.83). However, the proportions of HPV18 infection in both subgroups was similar (OR = 1.78, 95%CI 0.61–5.28). In the low-grade lesions group, non HPV16/18 infections were more popular than that in high-grade lesions group (OR = 3.04, 95%CI 1.59–5.82) (Table 3).

**Table 3:** Comparison between two subgroups of the multicentric lesion group

		Low-grade lesions group, *n* = 208 (%)	High-grade lesions group, *n* = 97, (%)	OR(95% CI)
Age, median (Q1-Q3)		50 (43–56)	50 (41.5–55)	
The grade of vaginal and/or vulval lesion, n (%)	VAIN1/VIN1	111(53.4)	28(28.9)	2.82 (1.68–4.73)
	VAIN2–3/VIN2	97(46.6)	69(71.1)	
HPV test, n (%)	Positive	198(95.7)	94(100)	NA
	Negative	9(4.3)	0	
Multiple HPV infection, n (%)	Yes	43(37.1)	20(33.3)	0.85 (0.44–1.64)
	No	73(62.9)	40(66.7)	
HR-HPV viral load, n (%)	>1000	19(26.8)	8(27.6)	0.96 (0.36–2.53)
	≤1000	52(73.2)	21(72.4)	
No. of patients with available HPV genotype results, n		115	60	
HR-HPV genotype, n (%)	16	36(31.3) ^a^	32(53.3)	2.54 (1.34–4.83)
	18	8(7.0)	7(11.7)	1.78 (0.61–5.28)
	Non 16/18 genotypes	72(62.6)	21(35.0)	3.04 (1.59–5.82)

^a^ one patient had both HPV16 and HPV18 infection

*HPV* human papillomavirus, *HR-HPV* high-risk human papillomavirus, *OR* odds ratio, *CI* confidence interval

### Risk factors associated with multicentric lesions

In the univariable analysis (Table 1), multicentric lesions were found to be associated with several factors, including menopause (OR = 2.73, 95% CI 2.13–3.50), a history of malignant tumors beyond the lower genital tract (OR = 5.05, 95% CI 2.21–11.55), multiple HPV infections (OR = 1.54, 95% CI 1.09–2.17) and HR-HPV viral loads > 1000 RLU/CO (OR = 1.76, 95% CI 1.05–2.95).

In the multivariable analysis (Table 1), menopause (OR = 3.14, 95% CI 2.24–4.41), a history of malignant tumors (OR = 9.58, 95% CI 1.02–89.84) and multiple HPV infections (OR = 1.47, 95% CI 1.03–2.10) were found to be independent risk factors for multicentric lesions.

Correlations among the top seven common HR-HPV genotypes and multicentric lesions detection are shown in Table 4. Of the 52 patients with HPV51 infection, 17 patients (32.7%) were diagnosed with multicentric lesions on histopathology. The risk of detecting multicentric lesions in patients with HPV51 infections was 2.37 times greater than detecting them in patients with non-HPV51 infections (OR = 2.37, 95% CI 1.29–4.33). Accordingly, HPV53 and HPV56 infections were correlated with multicentric tumor detection (OR = 2.35, 95% CI 1.40–3.93; OR = 1.82, 95% CI 1.01–3.26, respectively)*.* After adjusting for menopause, smoking, Gravidity, Parity, A history of malignant tumor beyond the lower genital tract and multiple HPV infection, only HPV51 infection was correlated with multicentric lesion detection (OR = 1.97, 95% CI 1.04–3.75).

**Table 4:** The correlation between the top seven common high-risk HPV genotypes and multicentric lesions detection

HPV genotypes	No. of multicentric lesions, n (%)	Univariable analysis OR (95% CI)	Adjusting for menopause, smoking, gravidity and etc. * OR (95% CI)
HPV16, *n* = 407	69(17.0)	0.90 (0.65–1.26)	0.93 (0.66–1.31)
HPV53, *n* = 75	24(32.0)	2.35 (1.40–3.93)	1.61 (0.92–2.81)
HPV58, *n* = 137	22(16.1)	0.86 (0.53–1.41)	0.83 (0.50–1.39)
HPV52, *n* = 142	19(13.4)	0.68 (0.41–1.13)	0.56 (0.32–0.97)
HPV51, *n* = 52	17(32.7)	2.37 (1.29–4.33)	1.97 (1.04–3.75)
HPV56, *n* = 62	17(27.4)	1.82 (1.01–3.26)	1.59 (0.85–2.98)
HPV18, *n* = 84	16(19.0)	1.09 (0.62–1.93)	1.09 (0.60–1.98)

* adjusted for menopause, smoking, gravidity, parity, a history of malignant tumor beyond the lower genital tract and multiple HPV infection

HPV, human papillomavirus

## Discussion

In this retrospective case-control study, it was indicated that the risk factors for multicentric lesions included menopause, a history of malignant tumors beyond the lower genital tract and multiple HPV infections using the multivariable analyses. In the multicentric lesions group, the top seven common HPV genotypes were HPV16, HPV53, HPV58, HPV52, HPV51, HPV56 and HPV18. The risk of detecting multicentric lesions in patients with HPV51 infection was 1.97 times of that in patients with non-HPV51 infection adjusted for menopause, smoking, gravidity, parity, a history of malignant tumor beyond the lower genital tract and multiple HPV infection.

With the advancement of cervical cancer screening, and the standardization of the colposcopy procedure, the detection rate of vaginal or vulva intraepithelial neoplasia has gradually increased [9–11]. The incidence of multicentric lesions has been reported in only 4.4% of patients with CIN [3]. In recent years, He Y et al. conducted a prospective cohort study and reported that 19.4% of CIN patients had CIN combined with VAIN [12]. In this study, the proportion of patients with multicentric lesions detected synchronously were 15.7%. Thus, the incidence of multicentric lesions is increasing. Therefore, physicians should be aware that a patient with HPV infection could present with multicentric lesions.

The average age of patients with multicentric lesions has not been consistent in previous studies, ranged from 36.3 to 43 years [3–5, 13]. In this study, the median age of patients with multicentric lesions was higher than that reported in the previous literatures. This phenomenon might be due to different sample sizes, populations, or other factors. He Y et al. [12] reported that the occurrence of CIN with VAIN was increasing with the increase of age. Moreover, menopause was an independent risk factor for multicentric lesions in this study. On one hand, the average age of patients with VAIN or VIN reported in the literatures has generally been older than that of CIN patients [14–16]. On the other hand, perimenopausal period is one of the two infection peaks of HR-HPV [17, 18]. Due to the decrease of estrogen level, the vaginal epithelium becomes thinner and more prone to microscopic damage. Moreover, hormone fluctuations can cause immune system regulation disturbances, such as the level of IL-6 increasing and Th1 / Th2 balance shifting to Th1 [19, 20]. So, the elimination or inhibition of HPV infection is weakened during menopause or perimenopause [21].

Most patients with multicentric lesions had no clinical symptoms. VAIN mostly involved the fornix and the upper third of vagina, high-grade lesions even involved the whole vagina. The images of VAIN under colposcopy were mainly micropapillary hyperplasia, acetic acid white epithelium, punctate blood vessels and Lugol’s iodine non-coloring [11]. Frega et al. [5] compared patients with multicentric lesions detected synchronously with those detected sequentially, and found a significant correlation between immunosuppression and synchronously detected multicentric lesions. Hammer A et al. [22] provided molecular evidence of HPV latency in humans. And, immunosuppression can trigger the reactivation of latent papillomavirus genomes [23]. Moreover, in this study, a history of malignant tumor was a risk factor for multicentric lesions. Although the mechanism is still unclear, the immunity of patient with a history of malignant tumor might be suppressed due to the stress after surgery and the side effects of radiotherapy or chemotherapy.

Multicentric lesions development is closely related to HR-HPV infection in the lower genital tract; however, the pathogenesis is not completely clear. So far, there are three theories to the pathogenesis. First, one persistent infection with a certain HPV genotype could induce the development of several independent lesions, representing synchronous multicentric lesion development, where there are either high viral loads or a high-risk for transformation. Second, repeated independent infections with various HPV genotypes can induce independent lesions [1, 4]. Third, precancerous cell clones can spread in the lower genital tract and induce lesions occurring sequentially [2]. The detection of identical HPV integration sites supported this mechanism of clonally related lesions. Consistent with the first pathogenesis theory, in this study, the median HR-HPV viral load was significantly higher in the multicentric lesions group than in the single CIN group, and HR-HPV viral loads > 1000 RLU/CO were correlated with multicentric lesions with the univariable analysis*.* In addition, in high-grade lesions subgroup, more than half (53.3%) of the patients were HPV16 infection. Consistent with the second pathogenesis theory and previous study [5], in this study, multiple HPV infections were an independent risk factor for multicentric lesions. However, multiple HPV infections did not significantly increase the risk of high-grade lesion beyond that for the highest-risk type found [24]. In this study, the proportions of multiple HPV infections in both subgroups were similar.

The prevalence of HPV genotypes would be useful for the development of screening and management guidelines for multicentric lesions. In this study, HPV16 was also the most common genotype, especially in high-grade lesions subgroup; HPV53, HPV58, HPV52, HPV51 and HPV56 were all more common than HPV18. Similar findings have previously been reported. HPV18 was rare in Asian patients with VAIN [25]. A Japanese study reported HPV52, 16, 51, 53 and 56 were the most common types in patients with VAIN [26]. However, an Italian study [5] reported that the top four common HPV genotypes associated with multicentric lesions were HPV16, HPV18, HPV33 and HPV31. A study from 31 countries showed HPV18 was the second common genotype in VAIN2/3 [27]. The possible reasons for these inconsistencies are regional differences, different detection methods, different sample sizes. In this study, HPV53, HPV51, and HPV56 were more common in patients of the multicentric lesion group compared with those of the single CIN group. HPV51, HPV53 and HPV56 infections were correlated with multicentric lesion detection using logistic regression analyses. After adjusting for menopause status, a history of malignant tumors, and multiple HPV infections, only HPV51 infections were found to be a risk factor for multicentric lesion detection. Thus, we should be alert to the occurrence of multicentric lesions in patients with HPV51 infection. However, HPV51 pathogenesis needs to be further studied.

### Strengths and limitations of the study

This study was done in relatively higher sample size with appropriate analysis technique that provides important information regarding HPV genotypes prevalence and risk factors associated with multicentric lesions in northeastern China. However, this study had some limitations. First, the retrospective design of this study could miss some variables in the analysis such as data related to oral contraceptives and number of sexual partners. Second, the number of patients that had HPV genotyping performed was relatively limited. Hence, more studies, specifically large prospective studies, are required.

## Conclusions

Currently, the incidence of multicentric lesions is increasing. Multicentric lesions were closely related to menopause, a history of malignant tumors beyond the lower genital tract and multiple HPV infections. The common HPV genotypes were HPV16, HPV53, HPV58, HPV52, HPV51, HPV56 and HPV18. HPV51 infection was a risk factor for multicentric lesions detection. To avoid missing a diagnosis of multicentric lesions, comprehensive colposcopical evaluations of the cervix, vagina, and vulva is warranted especially in women with risk factors.

## Data Availability

The dataset analyzed in this study is available from the corresponding author upon reasonable request.

## Abbreviations

CIN: cervical intraepithelial neoplasia
VAIN: vaginal intraepithelial neoplasia
VIN: vulvar intraepithelial neoplasia
HPV: human papillomavirus
HR-HPV: high-risk human papillomavirus
OR: odds ratio
CI: confidence interval
